# Spanish Version of the System Usability Scale for the Assessment of Electronic Tools: Development and Validation

**DOI:** 10.2196/21161

**Published:** 2020-12-16

**Authors:** Magdalena Del Rocio Sevilla-Gonzalez, Lizbeth Moreno Loaeza, Laura Sofia Lazaro-Carrera, Brigette Bourguet Ramirez, Anabel Vázquez Rodríguez, María Luisa Peralta-Pedrero, Paloma Almeda-Valdes

**Affiliations:** 1 Clinical and Translational Epidemiology Unit Massachusetts General Hospital Boston, MA United States; 2 Department of Medicine Harvard Medical School Boston, MA United States; 3 Unidad de Investigación en Enfermedades Metabólicas Instituto Nacional de Nutrición Salvador Zubirán and Instituto Tecnológico y de Estudios Superiores de Monterrey Tec Salud Ciudad de México Mexico; 4 Facultad de Enfermería y Nutriología Universidad Autónoma de Chihuahua Chihuahua Mexico; 5 Centro Dermatológico Dr Ladislao de la Pascua Ciudad de México Mexico; 6 Departamento de Endocrinología y Metabolismo Instituto Nacional de Ciencias Médicas y Nutrición Salvador Zubirán Ciudad de México Mexico

**Keywords:** mHealth, usability, validation, System Usability Scale, Spanish

## Abstract

**Background:**

The System Usability Scale (SUS) is a common metric used to assess the usability of a system, and it was initially developed in English. The implementation of electronic systems for clinical counseling (eHealth and mobile health) is increasing worldwide. Therefore, tools are needed to evaluate these applications in the languages and regional contexts in which the electronic tools are developed.

**Objective:**

This study aims to translate, culturally adapt, and validate the original English version of the SUS into a Spanish version.

**Methods:**

The translation process included forward and backward translation. Forward translations were made by 2 native Spanish speakers who spoke English as their second language, and a backward translation was made by a native English speaker. The Spanish SUS questionnaire was validated by 10 experts in mobile app development. The face validity of the questionnaire was tested with 10 mobile phone users, and the reliability testing was conducted among 88 electronic application users.

**Results:**

The content validity index of the new Spanish SUS was good, as indicated by a rating of 0.92 for the relevance of the items. The questionnaire was easy to understand, based on a face validity index of 0.94. The Cronbach α was .812 (95% CI 0.748-0.866; *P*<.001).

**Conclusions:**

The new Spanish SUS questionnaire is a valid and reliable tool to assess the usability of electronic tools among Spanish-speaking users.

## Introduction

The mobile health (mHealth) concept encompasses clinical and public health practices that incorporate mobile devices, such as smartphones, tablets, personal digital assistants, and patient monitoring devices [1]. According to estimates, there are more than 325,000 mHealth apps for the most popular mobile platforms, iOS and Android [2]. Categories of mHealth products encompass monitoring, treatment, diagnosis, health professional support, well-being, health surveillance support, and health care administration [3]. Although download indexes and apps in the market have increased in the last 5 years, clinicians, researchers, and patients remain skeptical about the reliability of the data generated [4]. These limitations have led to a lack of knowledge regarding the efficiency, efficacy, and safety associated with mobile app utilization in clinical practice. Furthermore, health organizations recommend making assessments before software implementation to ensure safety and accurate data quality [5].

Usability is an essential part of software development and is commonly evaluated through questionnaires [4,6]. Questionnaires reflect users’ opinions and have the advantages of low cost, easy execution, and lack of necessary test equipment. Usability can be defined as the extent to which a product can be used by specific users to achieve specified goals effectively and efficiently while providing user satisfaction in a specific context of use (user technology interface) [7]. Due to the high demand for mHealth apps, usability evaluations are insufficient. Therefore, it is necessary to implement a usability metric that is context driven and standardized to efficiently assess clinically related software. There is no usability questionnaire specifically designed for mHealth apps. Previous studies have investigated usability models for mobile apps and have also modified existing usability questionnaires for use in mobile app usability studies [8].

The System Usability Scale (SUS) proposed by Brooke [9] in 1986 is a widely used questionnaire to assess the usability of a system, such as standard operating system–based software interfaces, webpages, and mobile apps. It has been implemented in several mHealth fields, including mental health (n=12), cancer (n=10), nutrition (n=10), pediatrics (n=9), diabetes (n=9), telemedicine (n=8), cardiovascular disease (n=6), HIV (n=4), sanitary information systems (n=4), and smoking (n=4) [2]. The SUS questionnaire has been translated into several languages, such as Portuguese [10], Indonesian [11], and more recently, Malay [12]. All translated versions have shown similar internal reliability compared with the original English version. Although there is a Spanish version [13], there is no evidence of the validity and reliability process. Therefore, it is necessary to have a Spanish version of the SUS that documents the validation process in order to guarantee the quality of the resulting questionnaire. The objective of this study is to develop and validate a Spanish version of the original English SUS, guaranteeing conceptual, semantic, and contextual equivalence between both questionnaires.

## Methods

### SUS Scale

The SUS scale is a 10-item questionnaire scored on a 5-point Likert-type scale from 1 (strongly disagree) to 5 (strongly agree). Its advantages include versatility, simplicity, low cost, accuracy, and validity. Its reliability (Cronbach α=.85) has been reported [11,12,14-16]. The questionnaire is designed to be answered after the user’s interaction with the system. It is arranged to alternate between positive and negative statements to avoid habitual bias from the respondent. The score contribution for the odd items (the positive statements) is the scale position minus 1 and the contribution for the even items (the negative statements) is 5 minus the scale position. The overall score is calculated from the sum of all item scores multiplied by 2.5, with the overall score ranging from 0 to 100. A system with a score above 85 is considered to have excellent usability, whereas a system with a score between 68 and 84 is considered to have good usability.

### Translation

The original SUS questionnaire was translated into a new Spanish version using the methodology described by Ortiz-Gutiérrez and Cruz-Avelar [17], following the international guidelines proposed by the World Health Organization [5] to ensure the semantic equivalence, quality, and consistency of meaning with the original version. The methodology included 9 steps: (1) preparation, (2) forward translation, (3) synthesis, (4) back translation, (5) review of the back translation, (6) revision of the target language phrasing, (7) harmonization, (8) piloting, and (9) completion.

First, for the preparation, we evaluated the measurement properties of the original tool, identifying differences and similarities among them. The author’s permission was requested to work with the scale.

Second, to achieve the forward translation, the original version of the SUS was translated into Spanish by 2 independent translators with an adequate understanding of the source language: one individual had a master’s degree in translation studies and the other was a professional certified in English language and linguistics whose native language was Spanish. Each of the 2 translators provided their own translated version in Spanish. The translators were blind regarding the usage of the tool. Both translations were compared by the working group to combine them into one preliminary version.

Third, the working group for the synthesis was composed of 5 health professionals who were native Spanish speakers. Two of them were research coordinators, another held a master’s degree in clinical epidemiology, and 2 had PhD degrees in clinical epidemiology. The team had knowledge and experience in clinical and epidemiological research. They compared both translations and adjusted them, focusing on semantic equivalence and language reliability, to obtain the first consensual version.

Fourth, for the back translation, the first consensual version of the new Spanish version of the SUS was translated into English by a native English speaker whose second language was Spanish to ensure its compatibility with the original English version. The translator was blind to the final use of the translation. The output was an English version of the SUS translated from the preliminary SUS Spanish version (Table S1 in Multimedia Appendix 1).

Fifth, we conducted the review of the back translation. The working group compared the translation of the reconciled version to the original version to assess the conceptual equivalence between the 2 versions.

Sixth, to achieve a revision of the target language phrasing, we revised the semantic equivalence and worked to improve the phrasing of the new Spanish SUS version. In this step, we intentionally checked the presence of double-negative statements and the usage of words that are easily understandable by a population of different backgrounds and educational attainments.

Seventh, for the harmonization step, all the translations produced during the process were reviewed to detect possible discrepancies and to obtain the prefinal version.

Eighth, the pilot was planned following the methodology described by Ortiz-Gutiérrez and Cruz-Avelar [17], ensuring similar and appropriate conditions for answering the questionnaire. Target participants for the piloting were part of a clinical trial that aimed to measure environmental exposure using electronic tools that was taking place at the Unidad de Investigación de Enfermedades Metabólicas at the Instituto Nacional de Ciencias Médicas y Nutrición Salvador Zubirán in Mexico City. All participants provided written informed consent, and the study was approved by the institution’s ethics committee. The study follows the principles of the Declaration of Helsinki.

Ninth, after the pilot data were collected, they were carefully analyzed to detect time spent, possible questions that emerged from the participants over the process, and semantic understanding during the usage.

### Validation and Reliability Process

The SUS questionnaire was validated for content validity, face validity, and reliability. The method for quantifying content validity was the content validity index (CVI) [18], which is based on expert relevance ratings. The questionnaire (Table S2 in Multimedia Appendix 1) was given to 10 mobile app developer experts, or computer system engineers who had been working on mobile app development for at least 3 years. They were asked to give a score from 1 (question not relevant to assess usability’s tool) to 4 (relevant question to assess usability’s tool) for the relevance of each item of the SUS questionnaire to assess the usability of an electronic tool. According to the method, scores of 3 and 4 were recategorized as 1 (relevant) and scores of 1 and 2 were recategorized as 0 (not relevant). The CVI was calculated for each item on the SUS questionnaire, and then the CVI average across items was calculated.

The face validity index (FVI) aims to assess the clarity and comprehensibility of the translated items. This was performed by 10 users, who were asked to give a score from 1 (item not clear and not understandable) to 4 (item very clear and understandable) to assess the clarity and comprehensibility of the translated items of the SUS questionnaire. Scores of 3 and 4 were recategorized as 1 (clear and understandable) and scores of 1 and 2 were recategorized as 0 (not clear or understandable) [18]. The FVI was calculated for each item on the SUS questionnaire and then computed by calculating the scale average.

Reliability testing was conducted with 88 respondents, based on the minimum sample size estimation to assess internal consistency. The sample size was computed according to the Cronbach α estimation [19] by considering an α of .70 with a precision of 0.10 and a 2-tailed significance level of .05 for 10 items. The sample size required was 82 participants. For the reliability testing, we invited participants aged 18 to 75 years who had used Zoom (Zoom Video Communications) at least twice over the last month. We selected Zoom because it is a widely known application that can be used on different electronic devices, such as cell phones, laptops, and tablets, covering different modalities of a system. The respondents were asked to use the SUS to assess the usability of Zoom. All the surveys were conducted using Google Forms. The URL was sent through WhatsApp to each participant.

The reliability analysis was computed using Cronbach α, a measure of internal consistency. A coefficient of .70 or higher is considered acceptable for internal reliability [20]. Statistical analysis was performed using IBM SPSS Statistics version 21.0 for Macintosh (IBM Corp).

## Results

After reviewing all translated versions, we re-evaluated the complete questionnaire to ensure the syntax and grammar had meaning as a whole. In the back translation, the most important differences from the original version were the terms “technical person” and “cumbersome,” since the literal translations in Spanish are different from the conceptual meaning. We considered it appropriate for this translation to use “*personal experto*,” and “*tedioso*,” respectively. Likewise, the word “system” was changed to the Spanish words for “electronic tools,” namely “*herramienta*,” as this version attempts to determine the usability of mobile apps and websites. The output of this step was a preliminary version of the new Spanish SUS version.

The pilot study was conducted with 10 users who answered the questionnaire in person after using a website to record diet and exercise. The time spent to answer the questionnaire was 10 to 12 minutes. The pilot data were carefully analyzed by the working group. A total of 3 questions—numbers 2, 5, and 9—were difficult to understand for most users due to the use of complex words. The misunderstood words were changed for synonyms such as “*funciones*,” “*compleja*,” and “*confiado*,” which made the questionnaire easier to understand.

The output of the translation process was a questionnaire of 10 items in Spanish, equivalent to the SUS version in English, that measures the usability of electronic tools (Multimedia Appendix 2). 

The CVI (Table 1) and FVI (Table 2) of the new Spanish version of the SUS were calculated to be 0.92 and 0.94, respectively. CVI and FVI scores above 0.80 for both tests indicates that all items in the questionnaire are relevant to the domain, clear, and comprehensible to experts and users.

**Table 1:** Content validity index based on the rating of the relevancy of items by 10 experts. The content validity index average was 0.92.

Item No	E1^a^	E2	E3	E4	E5	E6	E7	E8	E9	E10	I-CVI^b^
1	3	3	4	2	3	3	3	4	3	3	0.9
2	2	3	4	4	4	4	4	1	4	4	0.8
3	4	3	4	4	4	4	4	4	4	4	1
4	4	3	4	3	3	4	3	4	4	3	1
5	3	3	4	4	3	4	3	4	2	4	0.9
6	3	3	4	3	3	2	2	4	3	3	0.8
7	3	4	4	2	4	3	3	4	4	4	0.9
8	4	4	4	3	2	3	3	4	4	4	0.9
9	4	4	4	4	3	3	4	4	4	4	1
10	3	4	4	4	4	3	4	4	3	4	1

^a^E: expert.

^b^I-CVI: item content validity index.

**Table 2:** Face validity index based on the rating of the items’ clarity and comprehensibility by 10 target users. The face validity index average was 0.94.

Item No	U1^a^	U2	U3	U4	U5	U6	U7	U8	U9	U10	I-FVI^b^
1	4	4	4	4	4	4	3	4	3	2	0.9
2	3	3	4	4	3	2	4	4	3	4	0.9
3	4	4	4	4	4	4	4	4	4	3	1
4	4	3	4	4	4	4	3	4	4	4	1
5	3	2	4	4	4	3	3	3	4	3	0.9
6	3	3	4	4	4	2	4	3	4	3	0.9
7	3	4	4	4	4	4	4	3	2	3	0.9
8	3	4	4	4	4	4	4	3	4	3	1
9	4	4	4	4	4	4	4	4	4	3	1
10	4	4	4	4	4	4	2	3	4	4	0.9

^a^U: user.

^b^I-FVI: item face validity index.

The reliability testing was conducted using 88 users. Table S3 in Multimedia Appendix 1 shows the characteristics of the users. The average age was 32.5 years. Most of the users were of middle socioeconomic status and had a bachelor’s degree.

The Cronbach α for the new Spanish version of the SUS was .812 (95% CI 0.748-0.866; *P<*.001). This α value indicates the high internal reliability of the new questionnaire (Table 3). The final version was shared with the authors.

**Table 3:** Internal consistency of the total item statistics.

Item No	Scale mean if item deleted	Scale variance if item deleted	Corrected item total correlation	Cronbach α if item deleted
1	17.42	36.98	0.403	.806
2	17.67	35.90	0.495	.795
3	17.98	35.11	0.660	.778
4	18.10	41.33	0.129	.831
5	17.57	37.07	0.492	.796
6	17.62	34.62	0.602	.783
7	17.97	37.67	0.487	.797
8	17.93	34.63	0.610	.782
9	17.71	35.07	0.528	.792
10	18.04	36.43	0.538	.791

## Discussion

In this study, a Spanish version of the SUS questionnaire was developed and validated. The results of the validation process indicate that the elements were easy to understand and there were no semantic or content-related problems. The translated items were considered equivalent to the original version; therefore, the Spanish questionnaire is a reliable tool to assess the usability of tools for Spanish-speaking users.

Spanish is the native language of most countries in Latin America and the second most widely spoken native language in the world, with more than 400 million speakers. In addition, it is important to develop multilingual strategies to assess each new electronic tool for health research with a wide array of individuals. Although there is a Spanish version of the SUS scale in existence, to our knowledge the translation process is not documented and there is no information about its validity and reliability.

Similarly, some broad concepts of the first Spanish translation make adaptation difficult for current mobile software and websites. With the advent of mobile apps and websites for research proposes in Spanish-speaking countries and around the world, is necessary to develop tools with supporting local evidence to evaluate specifications of new devices to ensure the data collected are accurate to the user. However, the development of new tools requires additional cost and time. Therefore, adapting available questionnaires into other languages and ensuring their validity is the best alternative.

The new SUS scale in Spanish will allow researchers and clinicians to evaluate a Spanish tool’s usability in an accurate, practical, and low-cost manner. In our study, the questionnaire was proven to be easy to comprehend and apply.

For this study, we applied the methodology proposed by Ortiz-Gutiérrez and Cruz-Avelar [17], which is consistent with the guidelines of the World Health Organization [5]. This methodology was combined with the process reported by Mohamad Marzuki et al [13] in 2018, who translated the same tool to Malay. Among the strengths of this methodology, the planning of each of the steps of the process particularly enhanced the quality of the translation.

Only young adults were included in the study. Therefore, the applicability to other age ranges may be questioned. In addition, the representativeness of the sample in reflecting the rest of Latin America may need further studies, as results might vary by region. Although only individuals of Mexico City were included, Mexico City constitutes an important representation of several states and regions of the country, including the south, center, east, and west coast of Mexico. This characteristic makes it appropriate to carry out representative studies when the possibility to extend them to several regions across the country is limited.

In conclusion, the new Spanish version of the SUS is a valid and reliable version of the original English version, adapted to be used for electronic tools in clinical and health research settings.

## Appendix

Multimedia Appendix 1 Supplementary tables.

Multimedia Appendix 2 Spanish version of the System Usability Scale.

## Abbreviations

CVI: content validity index
FVI: face validity index
mHealth: mobile health
SUS: System Usability Scale
