# Supplementary material for: Spanish Version of the System Usability Scale for the Assessment of Electronic Tools: Development and Validation
Source: JMIR Hum Factors. 2020 Dec 16;7(4):e21161. doi: 10.2196/21161 (PMC7773510; doi:10.2196/21161)
Supplement: Multimedia Appendix 1 [file humanfactors_v7i4e21161_app1.docx]

Table S1. Back translation output. Survey for User-Friendliness of Electronic Devices

|  | Strongly disagree |  |  |  | Strongly agree |
| --- | --- | --- | --- | --- | --- |
| I would be willing to use this electronic device frequently | 1 | 2 | 3 | 4 | 5 |
| I think that this electronic device is unnecessarily complicated | 1 | 2 | 3 | 4 | 5 |
| I felt that this electronic device is easy to use | 1 | 2 | 3 | 4 | 5 |
| I feel the need for an expert to help in order to use this electronic device | 1 | 2 | 3 | 4 | 5 |
| I felt that the diverse functions for this electronic device were well integrated | 1 | 2 | 3 | 4 | 5 |
| I felt that there were too many inconsistencies with the electronic device itself | 1 | 2 | 3 | 4 | 5 |
| I feel like most people would be able to easily learn how to use this electronic device | 1 | 2 | 3 | 4 | 5 |
| I feel like using this electronic device is too complicated | 1 | 2 | 3 | 4 | 5 |
| I felt confident in being able to use this electronic device | 1 | 2 | 3 | 4 | 5 |
| I had to take a lot of time to learn how to use this electronic device | 1 | 2 | 3 | 4 | 5 |

Table S2. Content validity survey applied to 10 mobile app developer experts

| Question | Pregunta | Question not relevant to assess usability’s tool |  |  | Relevant question to assess usability’s tool |
| --- | --- | --- | --- | --- | --- |
| I think that I would like to use this system frequently | Me gustaría usar esta herramienta frecuentemente. | 1 | 2 | 3 | 4 |
| I found the system unnecessarily complex | Considero que esta herramienta es innecesariamente compleja | 1 | 2 | 3 | 4 |
| I thought the system was easy to use | Considero que la herramienta es fácil de usar. | 1 | 2 | 3 | 4 |
| I think that I would need the support of a technical person to be able to use this system | Considero necesario el apoyo de personal experto para poder utilizar esta herramienta | 1 | 2 | 3 | 4 |
| I found the various functions in this system were well integrated | Considero que las funciones de la herramienta están bien integradas | 1 | 2 | 3 | 4 |
| I thought there was too much inconsistency in this system | Considero que la herramienta presenta muchas contradicciones | 1 | 2 | 3 | 4 |
| . I would imagine that most people would learn to use this system very quickly | Imagino que la mayoría de las personas aprenderían a usar esta herramienta rápidamente. | 1 | 2 | 3 | 4 |
| I found the system very cumbersome to use | Considero que el uso de esta herramienta es tedioso | 1 | 2 | 3 | 4 |
| I felt very confident using the system | Me sentí muy confiado al usar la herramienta | 1 | 2 | 3 | 4 |
| I needed to learn a lot of things before I could get going with this system | Necesité saber bastantes cosas antes de poder empezar a usar esta herramienta | 1 | 2 | 3 | 4 |

Table S3. Demographic characteristics of subjects for reliability testing (n=88)

| Characteristic | Value |
| --- | --- |
| Age (years), mean, SD | 32.48 ± 8.49 |
| Gender, female, n (%) | 57 (67.7%) |
| Highest education, n (%) | |
| High school diploma | 6 (6.8%) |
| Bachelor’s degree | 50 (56.8%) |
| Master’s degree | 32 (36.36%) |
| Socioeconomic status, n (%) | |
| Low | 4 (4.54%) |
| Medium | 77 (87.5%) |
| High | 7 (7.95%) |
