# Supplementary material for: Spanish Version of the System Usability Scale for the Assessment of Electronic Tools: Development and Validation
Source: JMIR Hum Factors. 2020 Dec 16;7(4):e21161. doi: 10.2196/21161 (PMC7773510; doi:10.2196/21161)
Supplement: Multimedia Appendix 2 [file humanfactors_v7i4e21161_app2.docx]

Spanish version of the System Usability Scale

For each statement please rate how much you agree or disagree with what it says. The scale is from 1 to 5. A rating of 1 means that you strongly disagree with the statement and a rating of 5 would mean that you strongly agree with the statement. A rating of 3 would mean that you are neutral about the statement.

Por favor seleccione de cada uno de los enunciados la opción que mejor describa su experiencia con la herramienta electrónica. Un puntaje de 1 significa que usted se encuentra totalmente en desacuerdo con el enunciado, mientras que un puntaje en 5 significa que está totalmente de acuerdo, un puntaje de 3 significaría que usted se encuentra neutral con el enunciado.

| No. | Question | Pregunta | Scale | | | | |
| --- | --- | --- | --- | --- | --- | --- | --- |
|  |  |  | Strongly disagree  [Totalmente en desacuerdo] |  |  |  | Strongly agree [Totalmente de acuerdo] |
| 1 | I think that I would like to use this system frequently | Me gustaría usar esta herramienta frecuentemente. | 1 | 2 | 3 | 4 | 5 |
| 2 | I found the system unnecessarily complex | Considero que esta herramienta es innecesariamente compleja | 1 | 2 | 3 | 4 | 5 |
| 3 | I thought the system was easy to use | Considero que la herramienta es fácil de usar. | 1 | 2 | 3 | 4 | 5 |
| 4 | I think that I would need the support of a technical person to be able to use this system | Considero necesario el apoyo de personal experto para poder utilizar esta herramienta | 1 | 2 | 3 | 4 | 5 |
| 5 | I found the various functions in this system were well integrated | Considero que las funciones de la herramienta están bien integradas | 1 | 2 | 3 | 4 | 5 |
| 6 | I thought there was too much inconsistency in this system | Considero que la herramienta presenta muchas contradicciones | 1 | 2 | 3 | 4 | 5 |
| 7 | . I would imagine that most people would learn to use this system very quickly | Imagino que la mayoría de las personas aprenderían a usar esta herramienta rápidamente | 1 | 2 | 3 | 4 | 5 |
| 8 | I found the system very cumbersome to use | Considero que el uso de esta herramienta es tedioso | 1 | 2 | 3 | 4 | 5 |
| 9 | I felt very confident using the system | Me sentí muy confiado al usar la herramienta | 1 | 2 | 3 | 4 | 5 |
| 10 | I needed to learn a lot of things before I could get going with this system | Necesité saber bastantes cosas antes de poder empezar a usar esta herramienta | 1 | 2 | 3 | 4 | 5 |
